# Metal ions in biomedically relevant macromolecular structures

**DOI:** 10.3389/fchem.2024.1426211

**Published:** 2024-08-23

**Authors:** Karolina A. Majorek, Michal Gucwa, Krzysztof Murzyn, Wladek Minor

**Affiliations:** ^1^ Department of Molecular Physiology and Biological Physics, University of Virginia, Charlottesville, VA, United States; ^2^ Department of Computational Biophysics and Bioinformatics, Jagiellonian University, Krakow, Poland; ^3^ Doctoral School of Exact and Natural Sciences, Jagiellonian University, Krakow, Poland

**Keywords:** metalloproteins, metal-protein complexes, structural biology, reproducibility, biomedical research, drug discovery

## Abstract

Understanding the functions of metal ions in biological systems is crucial for many aspects of research, including deciphering their roles in diseases and potential therapeutic use. Structural information about the molecular or atomic details of these interactions, generated by methods like X-ray crystallography, cryo-electron microscopy, or nucleic magnetic resonance, frequently provides details that no other method can. As with any experimental method, they have inherent limitations that sometimes lead to an erroneous interpretation. This manuscript highlights different aspects of structural data available for metal-protein complexes. We examine the quality of modeling metal ion binding sites across different structure determination methods, where different kinds of errors stem from, and how they can impact correct interpretations and conclusions.

## 1 Introduction

Metal ions play a crucial role in many biological processes in organisms from all kingdoms of life (Holm et al., 1996; Harding et al., 2010; Sigel et al., 2015). They can form complexes with biological macromolecules, including proteins, nucleic acids, and carbohydrates. Many human proteins (30%–40%) are estimated to interact with metal ions in various capacities (Andreini et al., 2009; Loginova et al., 2019). Frequently, these interactions assist folding, stabilize the structure, or alter the conformation, consequently dictating the function of these molecules (Gomes and Wittung-Stafshede, 2016). Metal ions that reside in the active site of enzymes facilitate substrate binding and reaction catalysis. The specific properties of a metal ion, like its charge and coordination geometry, influence the enzyme’s activity and specificity. Similarly, metal ions, especially magnesium, are crucial for the folding, stability, regulation, and biological activity of nucleic acids (Misra and Draper, 1998). Metal ions play key roles in biomedical research, both in studies of physiological functions and medical applications (Moustakas, 2021). Understanding the detailed roles of metal ions in biological systems is crucial for developing new therapies and interventions. It is important to note that maintaining the balance of metal ion concentrations in the body is essential for overall health, as both deficiencies and excesses can lead to various health problems (Jomova et al., 2022). Many medications can affect ion absorption or utilization, leading to health issues. Understanding how metal ions contribute to disease progression can offer clues for potential interventions. A better understanding of metal ion-binding proteins in diseases can help develop diagnostic tools and therapeutic strategies (Jomova et al., 2022).

Structural data deposited in the Protein Data Bank (PDB) (Berman et al., 2000; Berman et al., 2020; Burley et al., 2017) is one of the best resources for understanding the interactions of metal ions with biological macromolecules at the atomic level. PDB is an invaluable resource with over 70 thousand structures containing metal ions. Analyzing this data effectively requires significant experience and knowledge. Structures deposited in the PDB have inherent limitations and can also contain errors, leading to incorrect interpretations and conclusions. In this manuscript, we discuss various aspects of metal ion complexes within the PDB, illuminate exciting avenues for exploration, and indicate data quality shortcomings.

## 2 Roles of metal ions in various aspects of biology and medicine

Many metalloproteins contain metal ions as integral components, while others bind them transiently in cellular processes like transport and signaling. Ions of metals like magnesium, iron, zinc, and copper are crucial components of enzymes, stabilizing their structure and providing their biological function, and each of them also plays multiple other roles in the body (Jomova et al., 2022).

Calcium (Ca^2+^) is the most abundant metal in the human body, most often associated with skeletal health, but it is also involved in muscle function, nerve transmission, and enzyme activity. Magnesium (Mg^2+^) is also a cofactor in more than 300 enzymatic reactions and a multitude of cellular processes (Jahnen-Dechent and Ketteler, 2012). Working in concert, calcium and magnesium are essential for proper muscle contraction and relaxation (Potter et al., 1981), optimal nerve transmission and neuromuscular coordination (Kirkland et al., 2018), bone mineralization, and maintenance of normal bone (Rondanelli et al., 2021). It has been shown that stress can increase magnesium loss, and in turn, magnesium deficiency can further enhance susceptibility to stress, resulting in a magnesium and stress vicious circle (Pickering et al., 2020). Magnesium is also of interest for the potential prevention and treatment of numerous neurological disorders (Kirkland et al., 2018), sleep disorders (Cao et al., 2018), type 2 diabetes (Barbagallo and Dominguez, 2015), hypertension and cardiovascular disease (Houston, 2011). Magnesium is crucial in nucleic acid interactions, playing a key role in RNA folding (Misra and Draper, 1998; Misra and Draper, 1002) as well as DNA integrity and overall genomic stability (Hartwig, 2001; Anastassopoulou and Theophanides, 2002).

Iron, while essential for transporting oxygen throughout the body as a central component of hemoglobin, also plays a critical role in energy production in the mitochondria as a component of cytochromes. Methemalbumin is an albumin complex consisting of albumin and heme. Its presence in plasma is a diagnostic marker to differentiate between hemorrhagic and edematous pancreatitis. Thyroid peroxidase, an enzyme responsible for synthesizing thyroid hormones, also requires iron for its enzymatic activity. Therefore, iron deficiency anemia can impair thyroid function (Hess et al., 2002). Iron status is also associated with mood, cognition, and functional ability in older adults (Portugal-Nunes et al., 2020).

Zinc plays a vital role in numerous bodily functions across various systems, like growth and cell proliferation, DNA synthesis (MacDonald, 2000), protein synthesis (Kimball et al., 1995), lipid and glucose metabolism (Olechnowicz et al., 2018), wound healing (Lin et al., 2018), numerous aspects of immune system function (Prasad, 2008), and more. Recent studies (Wu et al., 2024) reveal that the binding of zinc ion (Zn^2+^) to human serum albumin affects fatty acid metabolism by inducing allosteric structural rearrangements at the interface between two protein domains.

Despite being present in the body at much lower concentrations, other metal ions also play critical roles in various processes. For example, copper is involved in primary metabolism, and aerobic life depends on its homeostasis and distribution (Peña et al., 1999). It is a crucial component of cytochrome *c* oxidase, an enzyme essential for the final step of cellular respiration – energy production. But copper is also a component of superoxide dismutase (together with zinc) that protects cells from damage caused by reducing oxidative stress and plays a role in various diseases (Ferenci, 2005; Lewandowski et al., 2019). Copper is also essential in iron metabolism (Collins et al., 2010), connective tissue formation (Harris et al., 1980) and development and function of the brain (Lutsenko et al., 2019).

Metal ions and their compounds have several unique characteristics that make them particularly well-suited as scaffolds for innovative drugs and diagnostic tools. They can exist in various oxidation states and can adopt diverse geometries and coordination numbers. They also maintain physiologically relevant redox states and can bind to a diverse range of organic molecules. These properties collectively enhance their versatility in medical and diagnostic applications (Yousuf et al., 2021).

Metal ions have been used in medicine for centuries. Dating back to around 1500 BCE, the Ebers Papyrus, an Egyptian medical record, provides a glimpse into one of the earliest documented uses of metals for medicinal purposes. The text mentions treatments like copper for headaches and iron-rich meat applied to wounds. Antimicrobial use of copper has been known for centuries, from sanitization of drinking water to treating wounds; it was used by ancient Egyptians, Greeks, and Romans (Arendsen et al., 2019). Processed metals including mercury, gold, silver, lead, zinc, and copper were used as therapeutics in ancient India (Galib et al., 2011). As a more recent example, in 1929, Jacques Forestier discovered that gold salt injections could alleviate joint pain and even induce remission in rheumatoid arthritis (Forestier, 1932). This led to widespread use of gold therapy, known as chrysotherapy, until the 1990s, when the development of safer and more effective treatments eventually led to its decline (Balfourier et al., 2020).

Modern medicine utilizes numerous metals in sophisticated diagnostic equipment to examine tissues and processes in previously unimaged ways. Gadolinium-based MRI contrast agents are the best-known example of metal ions use in diagnostics (Kanal et al., 2024). Manganese (Mn^2+^) acts as a contrast agent in functional MRI scans and PET imaging (Brandt et al., 2019). Due to similarities between Mn^2+^ and calcium (Ca^2+^), the former is used in manganese-enhanced MRI (MEMRI) technique, as it may enter neurons through voltage-gated Ca^2+^ channels and be used to trace neuronal pathways, define morphological boundaries, and study connectivity in morphological and functional imaging studies (Malheiros et al., 2015). Technetium-99m (Tc-99m) is a radioactive isotope commonly used in various diagnostic scans, including bone scans (detecting fractures, infections, or tumors), myocardial perfusion imaging (assessing heart function), thyroid scans (evaluating thyroid function) and brain scans (detecting abnormalities like infections or tumors) (Duatti, 2021). Barium sulfate is a common contrast agent used in X-ray imaging of the gastrointestinal tract (Martin-Harris et al., 2020).

Recently, platinum-based compounds have been widely used to treat various cancers by inducing DNA damage in cancer cells. The success of platinum-based therapies in cancer treatment comes at a cost: significant side effects, toxicity, and the emergence of drug resistance. Since the discovery and clinical applications of cisplatin (approved by FDA in 1978), other metal-containing scaffolds have been heavily explored as anti-cancer agents, utilizing metals like platinum, ruthenium, iridium, copper, gold, and osmium (Lucaciu et al., 2022; Adhikari et al., 2024). Next generations of platinum-based compounds, with lower toxicity and better efficacy than cisplatin, continue to be the most extensively used; however, ruthenium stands out as a promising alternative due to its lower toxicity and effectiveness against drug-resistant cancers (Lee et al., 2020; D’Amato et al., 2023; Kanaoujiya et al., 2023).

Abnormally high concentrations of metal ions, like copper, iron, and zinc, were found in amyloid plaques of Alzheimer’s disease patients (Abelein, 2023). Therapeutic chelation is being explored for the treatment and prevention of neurogenerative diseases; however, it still poses difficulties, like the toxicity of certain chelates and interference with physiological processes (Fasae et al., 2021). Diruthenium compounds with unique electronic and magnetic properties have recently been shown to act as inhibitors of amyloid aggregation, highlighting potential avenues in drug development for Alzheimer’s disease and novel application of bimetallic ruthenium-based drugs (La Manna et al., 2024).

Although numerous metal ion based drugs are currently utilized and investigated, there are many avenues that need further exploration. Coordination complexes exhibit a remarkable versatility due to their broad structural diversity, three-dimensionality, and tunable redox, catalytic, and photophysical properties. These inherent features increase their potential as a rich platform for the design of novel drugs (Karges et al., 2021). Catalytic metallodrugs, utilizing the catalytic potential of metal ions, represent a promising area of research, where the strategy is to catalyse the inactivation and degradation of therapeutic targets (Yu and Cowan, 2017).

## 3 Metal ions across different structure determination methods

**FIGURE 1:**
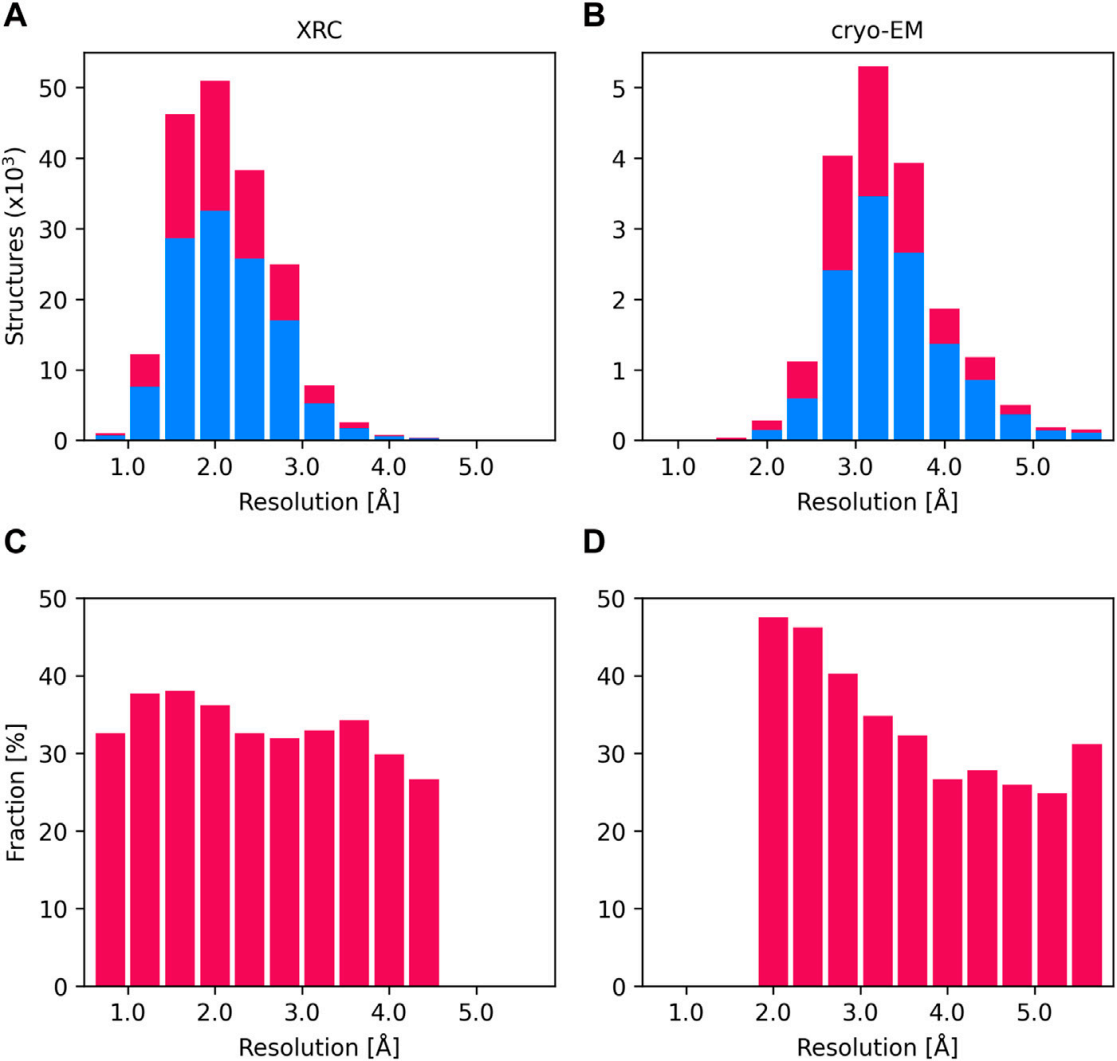
Distribution of the number of XRC structures **(A)** and cryo-EM structures **(B)** according to their resolution (intervals of 0.4 Ångström). The number of structures not containing metal ions (blue) and those containing at least one metal binding site (crimson) is displayed as stacked histograms. The total number of structures is the sum of deposits in these two categories. Charts **(C)** and **(D)** display the proportion of structures with metal ions as a fraction of all deposits, across each resolution interval, as identified by XRC and cryo-EM.

As of February 2024, approximately 33% of all protein structures in the PDB contain at least one metal ion (Figures 1A, B). Zn^2+^ and Mg^2+^ are the most frequently found in metalloprotein structures, followed by Ca^2+^ and iron (Fe^2+^ or Fe^3+^). About 35% of structures solved using X-ray crystallography (XRC) and 33% of structures solved by cryo-electron microscopy (cryo-EM) contain metal ions, while among NMR structures only 10% have metal ions bound. The fraction of deposited protein structures seems to be consistent across different resolutions in XRC (Figure 1C), while in cryo-EM with decreasing resolution we observe a decrease in the fraction of structures with metal ions (Figure 1D). This is likely because at low resolution in cryo-EM structures only the protein molecules are modeled, while solvent and ligands are usually impossible to identify and reliably model.

## 4 Overall reliability of modeling metal ion binding sites in the PDB deposits

To get a comprehensive picture of the modeled metal ion binding sites’ quality across PDB deposits we analyzed the metal ion-containing structures using CheckMyMetal (CMM) (Zheng et al., 2014; Zheng et al., 2017; Gucwa et al., 2023), which uses several key indicators to assess the quality of metal ion binding sites (MBS), including metal ion-ligand distances, completeness and geometry of coordination, and the agreement of B-factors with the ligand environment. (Zheng et al., 2008; Zheng et al., 2014).

**FIGURE 2:**
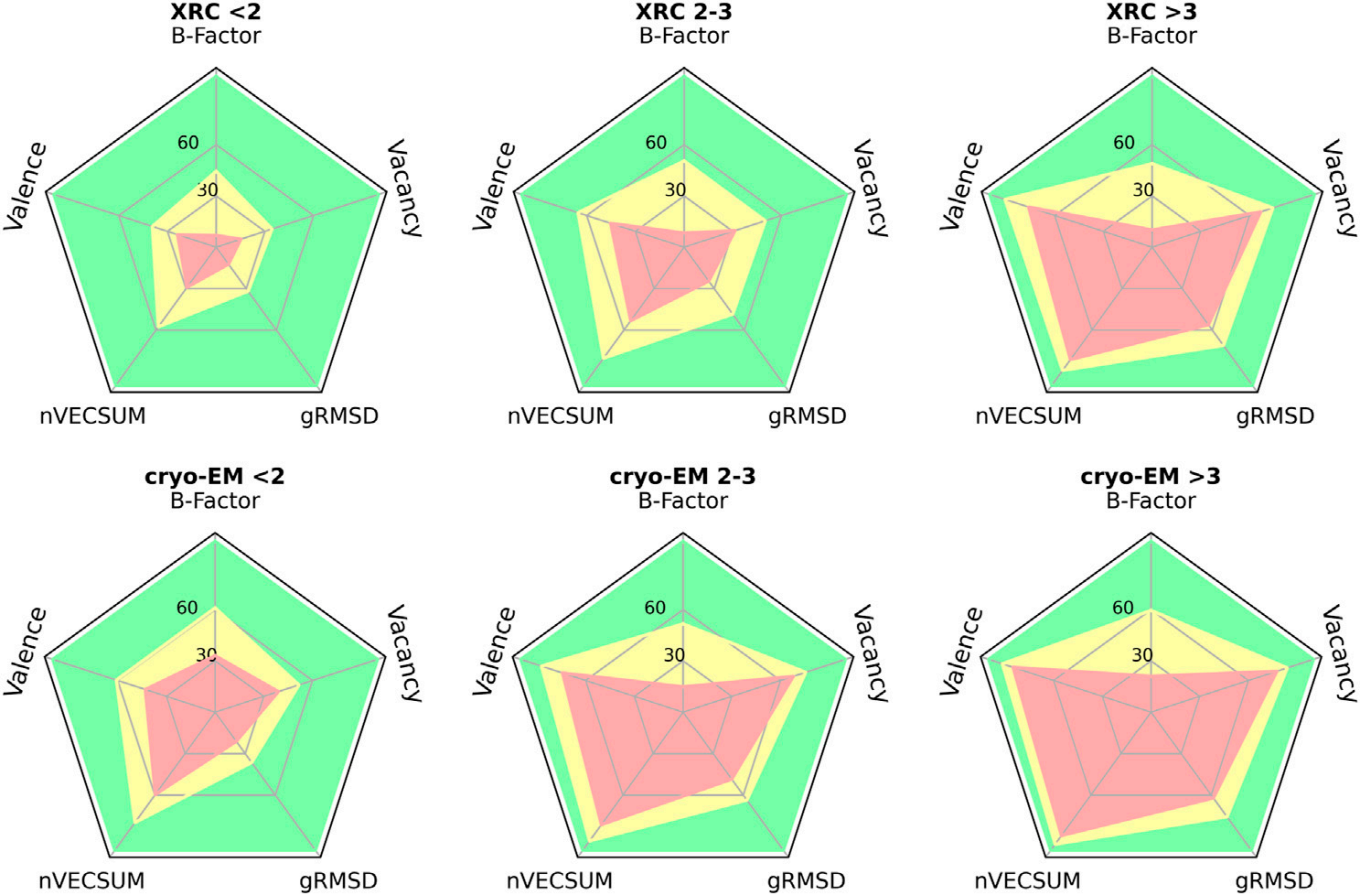
Radar plots for selected validation parameters of MBS in structures determined by XRC and cryo-EM in three distinct resolution ranges: below 2 Å, between 2 and 3 Å, and above 3 Å. The plots show the relative number of MBS, for which the validation parameter values correspond to one of three CMM categories: ACCEPTABLE (green), BORDERLINE (yellow), and DUBIOUS (red) - see (Zheng et al., 2014; Zheng et al., 2008; Gucwa et al., 2023) for details.

**FIGURE 3:**
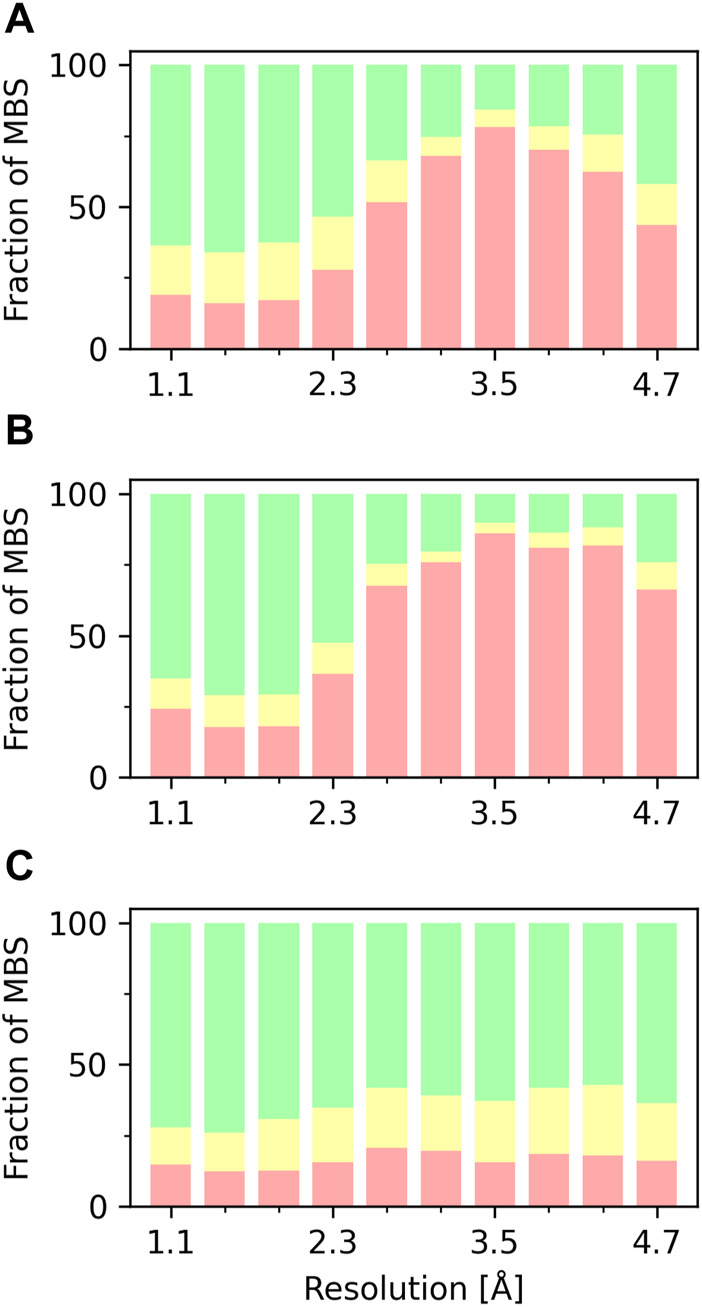
Fraction of MBS evaluated in CMM as ACCEPTABLE (green), BORDERLINE (yellow), and DUBIOUS (red), based on vacancy parameter, as a function of the XRC structure resolution. **(A)** MBS with any metal ion. **(B)** MBS with magnesium. **(C)** MBS with zinc.

The overall quality of the structural models deposited to PDB, including MBS is, unsurprisingly, correlated with data resolution (Figures 2, 3). At a resolution of ∼2.0 Å, the overall data quality is usually sufficient to model the coordination site reliably. The difficulty of interpreting the electron density map usually increases as the resolution deteriorates, which results in a relatively steep increase in poorly modeled or incomplete metal coordination sites. At low resolution (4.1–4.9 Å), we observe an improvement of the binding site model quality (Figure 3A). This may, at least partially, reflect the refinement strategies used for lower-resolution data. When the interpretation of electron density is more difficult, a higher weight is assigned to stereochemistry-based parameters. The quality of MBS is not always related to the overall quality of the structure. Therefore, a local evaluation of the sites of interest, rather than a global evaluation of the entire structures, may be a better approach (Yao and Moseley, 2019). Changes in a metal’s coordination sphere are unlikely to affect global metrics, like R-free or Clashscore, significantly, but they can drastically alter the metrics from CMM. The specific properties of each metal ion, like its charge and coordination geometry, influence the modeling of the binding site. Interactions between metal ions and protein are highly dynamic on the protein surface, where metal ions often interact both with conformationally flexible amino-acid residues and water molecules. Such a dynamic nature of these interactions presents increased challenges in structure modeling. Magnesium is an example of ion with more flexible coordination, therefore at lower resolutions the reliability of magnesium binding sites decreases (Figure 3B). Zinc ions, for example, have more rigid and less complex coordination geometry, which results in more straightforward interpretation of the electron density map; therefore, the fraction of acceptable zinc binding sites is consistently high across all resolution ranges (Figure 3C).

## 5 Metal ion misassignment

The poor ion binding indicators are frequently a result of problematic electron density and overall difficulty in the model building and refinement. However, this is not always the case. Numerous PDB deposits show incorrectly assigned metal ions in very well-defined binding sites that if properly examined by a knowledgeable researcher, or validated by a designated tool, could have been easily identified and errors could have been avoided.

**FIGURE 4:**
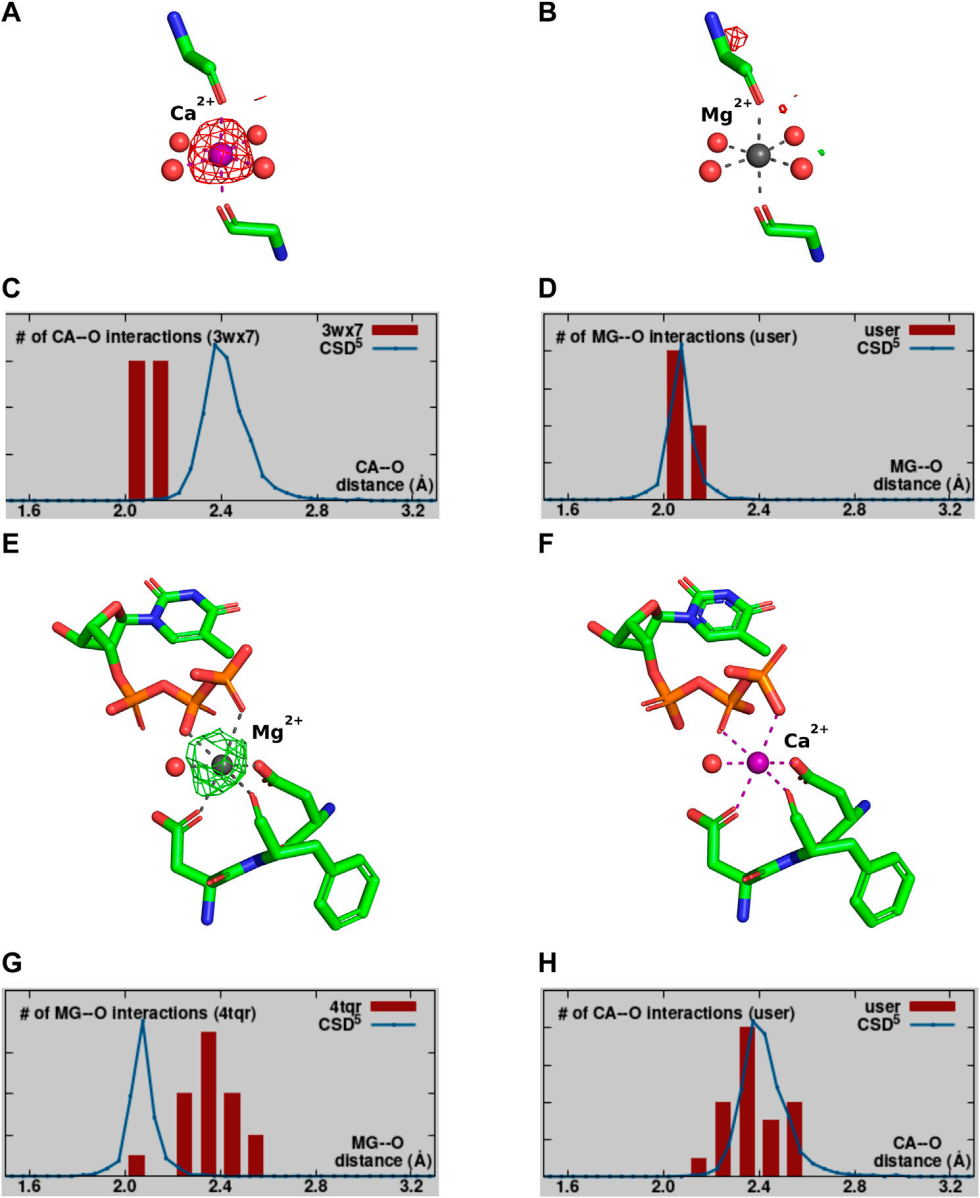
Example of a misassignment of metal identity. A-D PDB ID: 3WX7. **(A,B)** show the MBS with the Fo-Fc map surrounding the metal ion contoured at a sigma level of 3.0. **(A)** The MBS originally modeled with Ca^2+^. **(B)** The MBS re-refined with Mg^2+^. **(C,D)** The distribution of metal ion-ligand distances (histogram bars) as determined in CMM for MBS in **(A,B)**, respectively, compared against the appropriate data from the Cambridge Structural Database (Groom et al., 2016). E-H PDB ID:4TQR. Panels **(E,F)** show the MBS with the Fo-Fc map surrounding the metal ion contoured at a sigma level of 3.0. **(E)** The MBS originally modeled with Mg^2+^. **(F)** The MBS re-refined with Ca^2+^. **(G,H)** The distribution of metal ion-ligand distances (histogram bars) as determined in CMM for MBS in **(E,F)**, respectively.

Validation of the modeled metal ion should be considered a necessary step in the structure modeling process. The CMM (Zheng et al., 2014; Zheng et al., 2017; Gucwa et al., 2023) web server (https://cmm.minorlab.org/) provides an easy method to evaluate metal ion binding sites in macromolecular structures. CMM provides researchers with a number of parameters regarding the modeled metal, classifying them as acceptable, borderline, or dubious. In problematic cases, CMM suggests a metal ion that could be a better fit. In Figure 4 we show an example of a metal ion incorrectly assigned in a well-defined binding site. Originally modeled Ca^2+^ shows a negative electron density map (Figure 4A), which should have indicated to the researcher that the ion assignment is problematic. The distribution of bond lengths indicates these are not in the preferred range for the Ca^2+^ (Figure 4C) modeled by the researcher, and Mg^2+^ is suggested as a better fit. Mg^2+^ was also present in crystallization conditions. Based on the validation provided by CMM, the misassigned Ca^2+^ was replaced by Mg^2+^, which shows a much better fit in both geometry and electron density (Figures 4B, D).

Magnesium is by far the most common metal ion interacting with nucleic acids, this can lead to misleading assumption about its presence in the structure. Figures 4E–H shows an example, where Mg^2+^ was assumed to be bound, but the analysis of the binding site and re-refinement prove that Ca^2+^ (that was also present in the crystallization conditions) is more appropriate.

Additional difficulty in assigning a proper metal ion may also arise in the proteins which can bind more than one kind of metal ion in the same binding site. Serum albumin, which serves as a promiscuous transporter in blood, is an example of a protein that has this capability, and various metal ions compete for the same binding sites (Handing et al., 2016; Handing et al., 2018; Czub et al., 2019). Metal interchangeability is also an adaptation mechanism (Smethurst and Shcherbik, 2021). The metal incorporation into a macromolecular complex will be influenced by metal ion availability in particular physiological and environmental conditions. The possibility of different metal ions binding to the same sites will be limited by the size, charge, and chemical properties of specific ions, as well as the flexibility of the binding site (Smethurst and Shcherbik, 2021).

Water and some metal ions (especially lighter ones like sodium or magnesium) have similar electron scattering properties. This, especially in more flexible binding sites and at lower resolutions, where full coordination sphere may not be easily identified, can lead to interpreting the electron density as a water molecule instead of a metal ion. Since metal ions often play crucial roles in protein function and stability, mistaking them for water can lead to a flawed understanding of how the macromolecule works. Therefore, careful validation of metal or any other ligand is critical to ensuring the high quality and reliability of deposited macromolecular structures.

## 6 Metal ions occupancy

There are multiple modes of metal ion binding within proteins. Different binding strengths and life times can be observed, from transient interactions to strong, permanent complexes. Proteins might interact with metal ions transiently only under specific conditions, further complicating capturing such interactions. In the PDB, the fraction of molecules in the crystal that have a particular atom present at that specific position is referred to as occupancy. It is a value between 0 and 1, with 1 meaning all molecules in the crystal have the atom at that position, while less than 1 indicates partial occupancy, where some molecules lack the atom at that position. There may be several reasons for low occupancy in general. If the metal ion is weakly bound it may not be present in all protein molecules, thus the lower occupancy represents the fraction of molecules where the metal is bound. When the ion is located in a flexible region of the protein and lacks a well-defined structure, the low occupancy value indicates its mobility. A protein might have multiple binding sites for metal ion, with some chains favouring one over the other, leading to partial occupancy at specific sites. In rare cases, a very low occupancy might represent a biologically relevant subpopulation of the protein with a different metal ion bound.

**FIGURE 5:**
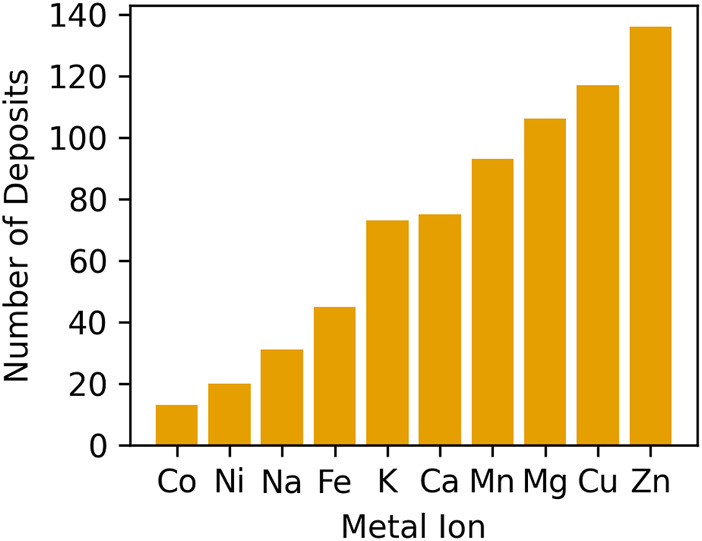
The number of PDB deposits that contain at least one metal ion with occupancy lower than 0.2.

**FIGURE 6:**
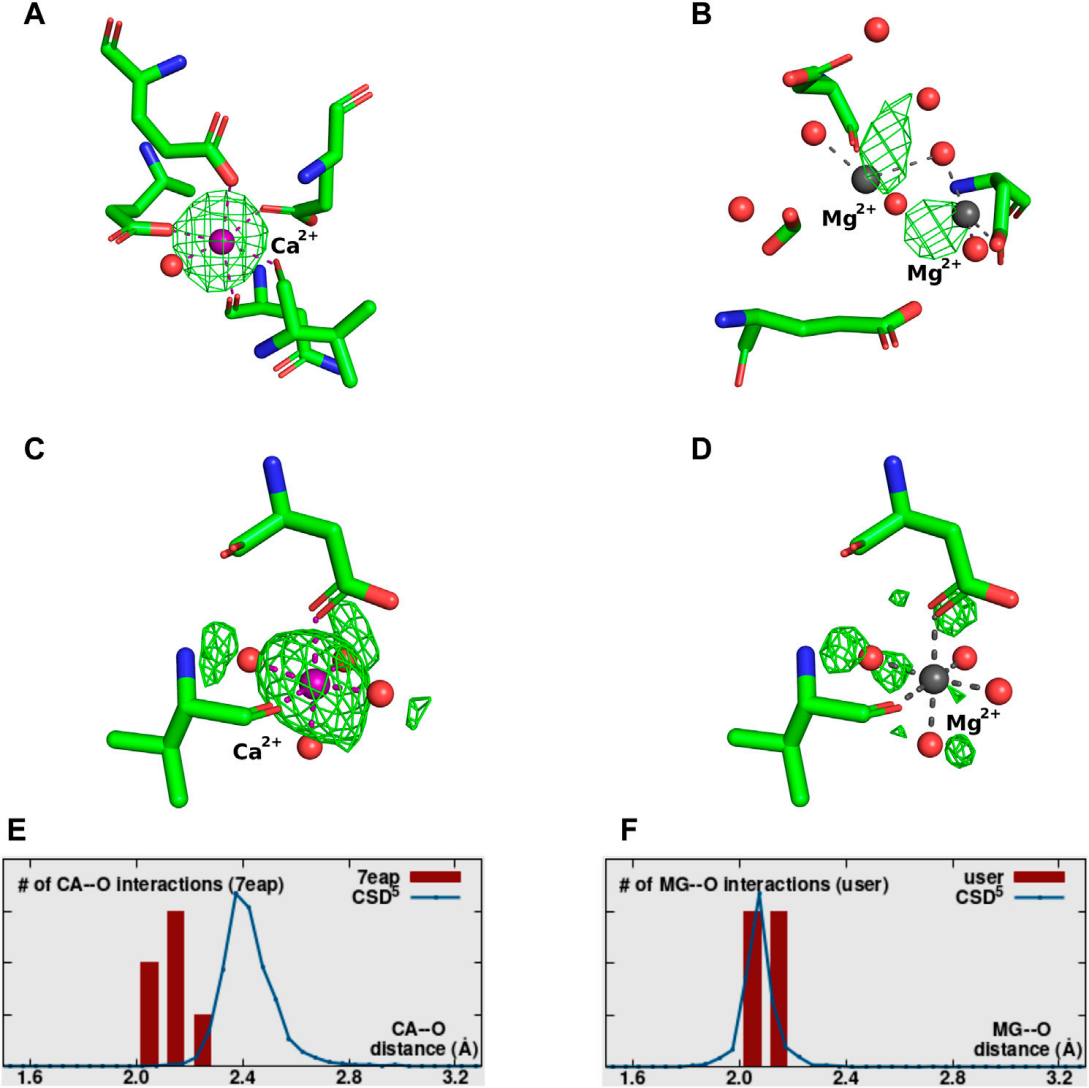
Examples of metal ions modeled with 0 occupancy. Each metal-binding site presents the Fo-Fc map contoured at a sigma level of 3.0. **(A)** 5XW9—correctly identified Ca^2+^ modeled with incorrect 0 occupancy. **(B)** 8H4A–incorrectly placed and identified 2 Mg^2+^ with 0 occupancy. **(C,D)** 7EAP–both panels correspond to the same metal-binding site. Panel C shows the site originally modeled with Ca^2+^ at 0 occupancy, while panel D shows the site re-refined with Mg^2+^ at 1 occupancy. **(E,F)** The distribution of metal ion-ligands distances (histogram bars) as determined by CMM for MBS in **(C,D)**, respectively, compared against the appropriate data from Cambridge Structural Database.

One of the inaccuracies we observe in the deposited structures of metalloproteins is unrealistically low occupancies of metal ions. About 700 structures in the PDB contain metal ions with occupancy below 0.2 (Figure 5). Among these, at least 72 magnesium ions, 42 zinc ions, and 36 calcium ions are modeled with occupancy below 0.1, and many of them with occupancy values of 0. Instances like this require careful interpretation. Zero occupancies would mean that the modeled entity is not there (hence should not be present in the model at all), yet in many cases, we observe the electron density map indicating the presence of such atom (Figure 6). These atoms are particularly problematic because even experienced structural biologists can overlook the low occupancy of an atom when looking at a structure in one of the molecular visualization programs.

Structure 5XW9 (Figure 6A) is an example of a situation when the metal ion has been modeled with occupancy 0, even though both 2Fo-Fc and a positive Fo-Fc electron density maps indicate it is present (and therefore should be modeled with non-zero occupancy value). In this case, placing the ion in the model is justified, but assigning zero occupancy is not. The structure 8H4A (Figure 6B), is an example where the position of some of the metal ions in the model is completely unjustified by the electron density map or coordination. Re-refining the structure with higher occupancy generates a negative Fo-Fc map, further confirming the incorrect placement of the ions. That should indicate to the researcher that the atom is not present at that position and should be removed from the model, or its position should be corrected.

In structure 7EAP (Figures 6C, E) not only was a Ca^2+^ ion modeled with an unjustified 0 occupancy, but the identity of the atom was also assigned incorrectly. Re-refinement with Mg^2+^ and occupancy 1 proves to be a much better fit (Figures 6D, F).

In some cases, the value might be used by a researcher consciously - the occupancy might be set to zero if information about an atom is missing due to experimental limitations, but a researcher “believes” it is there and decides to place it in the model. The problem with that approach is that many non-structural biologists use PDB as a source of information without fully understanding it and do not validate parameters like occupancy or quality of the electron density map. It is not sufficient to just look at the visualization of the model alone and believe it represents the actual state of the molecule.

Analysis of the surrounding residues, protein function, and known metal binding properties can help to assess the biological plausibility of low occupancy. However, even with supporting data from different characterization methods, the crystallographic data does not support the determination of instances of extremely low occupancies, hence these should be considered unreliable. It is therefore crucial to carefully evaluate the context and consider alternative explanations before drawing conclusions based on such low occupancy values.

## 7 Conclusion

Structural biology provides invaluable tools for biomedical research and drug discovery. The ability to determine protein structures with increasing accuracy and speed through XRC and cryo-EM is revolutionizing our understanding of biology and health. This information continues to fuel breakthroughs in drug discovery, disease diagnosis, and other scientific endeavours. While XRC and cryo-EM are powerful tools, inaccurate protein structure information can have a cascading effect, propagating errors and hindering research progress. Accurate protein structure information is not less crucial when considering metal ions bound within those structures. Identifying and accurately determining the coordination environment around a metal ion can be challenging, which can lead to misinterpretations of the metal’s role and function within the macromolecule.

While cryo-EM is currently a groundbreaking structure determination technique, the data interpretation can be very challenging due to the fact that the majority of cryo-EM structures have resolutions that are worse than XRC structures (Bijak et al., 2023). Frequently, the ideal geometrical parameters have higher weights during refinement, leading to idealized metal ion environment for poor resolution data (Shabalin et al., 2018). If that happens, without the use of complementary techniques or prior knowledge, a wrong metal ion can be assigned and modeled to “look good”, and errors can be easily missed (Raczynska et al., 2018; Wlodawer et al., 2018). In that respect, XRC has a few unique advantages (Table 1). First, XRC data provides us with difference maps, also known as Fo-Fc maps, which are a vital tool for researchers to refine and validate their proposed atomic models of a protein or molecule. These maps reveal the discrepancies between the experimental data and the model, helping scientists identify areas where their model might be inaccurate. As each metal has a unique electron configuration, different metal ions will have different electron densities, and will lead to presence of positive or negative difference map if modeled incorrectly. That should be the first indication that the researcher should validate the modeled ion. Another specialized XRC technique employs anomalous scattering (Ascone and Strange, 2009). The energy at which anomalous scattering occurs is unique for each element, particularly for transition metals commonly found in proteins. By tuning the X-ray wavelength to the diffraction edge of the anomalous scattering energy of a suspected metal ion, researchers can enhance the signal from that specific element within the crystal (Zheng et al., 2008). The ultimate assignment of metal ion can be achieved by performing two diffraction experiments, i.e., collecting data above and below the anomalous edge of the element under investigation (Zheng et al., 2014; Handing et al., 2018). Anomalous scattering can be especially beneficial to distinguish between metal ions with similar electron densities, for example, iron and manganese. Moreover, the anomalous signal significantly enhances the electron density in the difference map, providing an even clearer picture. While anomalous scattering has traditionally been used to solve the crystallographic phase problem, introducing the anomalous scatterers is not always feasible. The use of long-wavelength beamlines may be particularly beneficial in some cases by allowing even the small anomalous signals from sulfur or phosphorus to solve the phase problem. Certain biologically relevant metal ions, like calcium and potassium, also have absorption edges at longer wavelengths than is accessible at most beamlines. With long-wavelength beamlines, scientists can precisely tune the X-ray energy to match these lower-energy absorption edges, maximizing the metal ions’ anomalous signal.

**TABLE 1:** Summary of advantages and disadvantages of different structure determination methods in obtaining quality information for metal ion complexes.

X-ray crystallography	Cryo-electron microscopy
Pros:	Pros:
• Fo-Fc maps as a tool for metal ion and other ligands validation	• Close to native conditions
• Possible use of anomalous scattering at different wavelengths for metal identification	• Relative ease of sample preparation
• High-resolution data is frequently obtained and provides detailed information on ligand coordination geometry and distances	• If high-resolution data is obtained, it provides detailed information on ligand coordination geometry and distances
Cons:	Cons:
• Obtaining quality diffraction crystals is frequently a rate-limiting step	• Coulomb potential maps contain information about ion charge, but currently available methods cannot yet utilize that efficiently (Marques et al., 2019; Bick et al., 2024; Bochtler, 2024)
• Challenging interpretation at low resolution	• Challenging interpretation due to usually relatively low resolution
• Crystallization may potentially alter the protein structure and metal binding	

Considering that only in 2023, the daily average of downloads exceeded 8.5 million (https://www.wwpdb.org/stats/download), the deposition of structures to PDB became a foundation for further research. Errors in structures can mislead subsequent studies, creating a domino effect of wasted time and resources across many scientific fields. For example, previously known XRC structures are frequently used as templates for cryo-EM structure determination. Thus, incorrectly assigned metal ions may propagate to structures determined by cryo-EM and other techniques. Ensuring the quality of MBS for any downstream analysis and applications is critical for maintaining the reproducibility of biomedical research.

Acknowledging the potential for misinformation and implementing robust practices, researchers can ensure that protein structural data remains reliable for groundbreaking research.

## Abbreviations

XRC, X-ray crystallography; cryo-EM, cryo-electron microscopy; MBS, metal binding site; CMM, CheckMyMetal.
